# Personality and suicide risk: the impact of economic crisis in Japan

**DOI:** 10.1017/S0033291714001688

**Published:** 2014-07-18

**Authors:** F. Tanji, M. Kakizaki, Y. Sugawara, I. Watanabe, N. Nakaya, Y. Minami, A. Fukao, I. Tsuji

**Affiliations:** 1 Division of Epidemiology, Department of Public Health and Forensic Medicine, Tohoku University Graduate School of Medicine, Sendai, Miyagi, Japan; 2 Division of Community Health, Tohoku University Graduate School of Medicine, Sendai, Miyagi, Japan; 3 Department of Health Sciences, Tohoku University Graduate School of Medicine, Sendai, Miyagi, Japan; 4 Department of Preventive Medicine and Epidemiology, Tohoku Medical Megabank Organization, Tohoku University, Sendai, Miyagi, Japan; 5 Department of Public Health, Yamagata University Graduate School of Medical Science, Yamagata, Japan

**Keywords:** Cohort study, economic crisis, Japan, personality, suicide risk

## Abstract

**Background:**

The interactive effect of personal factors and social factors upon suicide risk is unclear. We conducted prospective cohort study to investigate whether the impact of the economic crisis in 1997–1998 upon suicide risk differed according to Neuroticism and Psychoticism personality traits.

**Methods:**

The Miyagi Cohort Study in Japan with a follow-up for 19 years from 1990 to 2008 has 29 432 subjects aged 40–64 years at baseline who completed a questionnaire about various health habits and the Japanese version of the Eysenck Personality Questionnaire – Revised Short Form in 1990.

**Results:**

The suicide mortality rate increased from 4.6 per 100 000 person-years before 1998 to 27.8 after 1998. Although both Neuroticism and Psychoticism were significantly associated with an increased risk of mortality during the whole period from 1990 to 2008, the impact of the economic crisis upon suicide risk differed between the Neuroticism and Psychoticism personality traits. Compared with the lowest category, the hazard ratios (HRs) for the highest Neuroticism increased from 0.66 before 1998 to 2.45 after 1998. On the other hand, the HRs for the highest Psychoticism decreased from 7.85 before 1998 to 2.05 after 1998.

**Conclusions:**

The impact of the 1997–1998 economic crisis upon suicide risk differed according to personality. Suicide risk increased among these with higher Neuroticism after the economic crisis, but this was not the case for other personality subscales.

## Introduction

Economic difficulty is one of the most important causes of suicide (Lewis & Sloggett, 1998; Inoue *et al.* 2007; Stuckler *et al.* 2009). The economic crisis in 1997–1998 harmed the economies of many Asian countries, and resulted in a sharp increase of suicide mortality (Chang *et al.* 2009; Kim *et al.* 2011). Until 1997 in Japan, the suicide rate had ranged from 16.1 to 18.8 per 100 000 people. However, in 1998, it jumped to 25.4, and since then has remained around 25.0 (Statistics and Information Department Minister's Secretariant Ministry of Health Labour and Welfare Japan, 2011).

The risk factors for suicide are not only economic, but also psychological, physical, and cultural (Bertolote & Fleischmann, 2005). Yoshimasu *et al.* (2008) divided these risk factors into two categories, personal and social factors, and they assumed that the interactive effects of these two factors may affect the suicidal risks.

Previous studies have agreed that personality traits such as Neuroticism and Psychoticism in the Eysenck Personality Questionnaire (EPQ) are associated with an increased risk of suicide (Lolas *et al.* 1991; Duberstein *et al.* 1994; Velting, 1999; Useda *et al.* 2004; Tsoh *et al.* 2005; Brezo *et al.* 2006). Since personality determines a person's response to life events (Kendler *et al.* 2004), we may hypothesize that the impact of economic difficulty upon suicide risk must differ according to personality traits. However, no study has investigated the interactive effects of economic difficulty and personality upon suicide risk.

The objective of this study was to investigate whether the relationship between personality and suicide differed before and after the economic crisis in 1997–1998. This knowledge would lead us to further understanding of the mechanisms how different personality traits cope with social stress, thus contributing to the planning of effective suicide prevention. For this purpose, we analysed data from a population-based prospective cohort study in Japan, which we have been following up since 1990. Our cohort is unique in that it can analyse the association between personality and suicide risk, both before the economic crisis (1990–1997) and after the crisis (1998–2008), separately.

## Methods

### Study cohort

The design of the Miyagi Cohort Study has been described in detail elsewhere (Fukaol *et al.* 1995; Nakaya *et al.* 2005). In brief, we delivered two self-administered questionnaires to all 51 921 residents (25 279 men and 26 642 women) aged 40–64 years in 14 municipalities of Miyagi Prefecture, northern Japan, between June and August 1990. The first questionnaire asked about various health habits, and the second was the Japanese version of the Eysenck Personality Questionnaire – Revised (EPQ-R) Short Form (Hosokawa & Ohyama, 1993). The questionnaires were delivered to, and collected from, the subjects’ residences by members of Health Promotion Committees appointed by the municipal government. We excluded one subject because of withdrawal before June 1990 (*n* = 51 920). The response rate for the first questionnaire was 91.7% (*n* = 47 604), and that for the second questionnaire among the respondents to the first was 87.0% (*n* = 41 423). The study protocol was approved by the institutional review board of Tohoku University School of Medicine. We considered the return of self-administered questionnaires signed by the participants to imply their consent to participate in the study.

### Exposure data

The EPQ-R has 48 questions with dichotomized responses (yes or no), and scores for each of the four subscales (Extraversion, Neuroticism, Psychoticism, Lie) are calculated on the basis of 12 questions each. The scores on each subscale range from 0 to 12, with higher scores indicating a greater tendency to possess the personality trait represented by each subscale. Extraversion represents sociability, liveliness and surgency; Neuroticism represents emotional instability and anxiousness; Psychoticism represents tough-mindedness, aggressiveness, coldness, and egocentricity; Lie represents unsophisticated dissimulation and social naivety or conformity (Eysenck & Eysenck, 1991).

Several of Eysenck's personality questionnaires have been translated into Japanease (Hosokawa & Ohyama, 1993). In previous work, Hosokawa *et al.* developed the Japanese version of the EPQ-R and examined its reproducibility and validity among 329 college students and 253 adults (Hosokawa & Ohyama, 1993). Cronbach's *α* coefficient, a measure of internal consistency, was >0.70 for all subscales except Psychoticism (0.42 for college students and 0.48 for adults). Test–retest reliability coefficients of the four subscales over a 6-month period ranged from 0.70 to 0.85, indicating substantial stability. Confirmatory factor analysis supported the original theoretical structure of the four scales proposed by Eysenck and colleagues.

### Follow-up

The endpoint was suicide mortality. To follow up the participants for mortality and migration, we established a Follow-up Committee (Tsuji *et al.* 2004). This Committee consisted of the Miyagi Cancer Society, the Community Health Divisions of all 14 municipalities, the Department of Health and Welfare of Miyagi Prefectural Government, and the Division of Epidemiology, Tohoku University Graduate School of Medicine. The Committee periodically reviewed the Residential Registration Record of each municipality. With this review, we identified participants who had either died or emigrated during the follow-up period. We discontinued follow-up of those who had emigrated from the study area, because the Committee could not review the Residential Registration Record outside the study area.

For decedents, we investigated the causes of death by reviewing the death certificates with permission from the Ministry of Health, Labour and Welfare, Japan. Cause of death was classified according to the International Classification of Diseases (ICD), 9th revision, between 1 June 1990 and 31 December 1998 (WHO, 1977), and 10th revision, between 1 January 1999 and 31 December 2008 (WHO, 1992). Death due to suicide was identified as ICD-9: E950-E959, or ICD-10: X60-X84.

Of the 41 423 subjects who responded to the two questionnaires, we excluded 54 subjects who responded ‘yes’ or ‘no’ to all 48 items and 8600 subjects for whom responses to any of the 48 items in the EPQ-R were missing. We also excluded 2493 subjects who indicated that the two questionnaires had been completed by other family members, because we considered that such aid might have affected the subjects’ response patterns. In addition, we excluded 844 subjects who had entered a history of cancer, stroke, or myocardial infarction in the self-reported questionnaire. Finally, 29 432 subjects (14 327 men and 15 105 women) were used for the final analysis.

We counted person-years of follow-up for each subject from 1 June 1990 until the date of death, the date of emigration from the study districts, or the end of follow-up, whichever occurred first. A total of 504 595 person-years resulted. Among the 29 432 subjects, the number of deaths due to suicide was 90 (suicide rate 17.8 per 100 000 people). A total of 1892 subjects (6.4% of the analytic cohort) were lost to follow-up during the study period.

### Statistical analysis

Each personality subscale was divided into four categories to distribute the total subjects as closely as possible into even-sized quartiles. We calculated the annual trend for the number of suicide deaths between 1990 and 2008 in the study subjects (*n* = 29 432). We used the Cox proportional hazards model to calculate the hazard ratios (HRs) and 95% confidence intervals (CIs) for suicide mortality according to each category of a personality subscale, the lowest category being treated as the reference group, and to adjust for potentially confounding variables. In the case of missing values for a confounding variable, we created a separate missing category and included this in the models. In addition, we conducted these analyses separately by sex (data not shown). Trend tests were performed by treating personality subscales as continuous variables.

In these analyses, we regarded the following variables as potential confounders: age (continuous variable), sex, history of hypertension or diabetes mellitus (presence or absence), smoking status (never, former, currently smoking 1–19 cigarettes/day, or ⩾20 cigarettes/day), alcohol consumption (never, former, current ethanol intake of <23.0, or ⩾23.0 g/day), body mass index in kg/m^2^ (⩽18.4, 18.5–24.9, or ⩾25.0), green tea consumption (<1 cup/day, 1–2 cups/day, or ⩾3 cups/day), education (in school until age £15 years, age 16–18 years, or age ⩾19 years), marital status (whether or not living with spouse), sleep duration (⩽6, 7–8, or ⩾9 h/day), time spent walking (⩽0.5, 0.5–1, or ⩾1 h/day), *ikigai* (having, uncertain, or not having), stress (high, moderate, or low).

In Japanese culture, having *ikigai* (sense of ‘life worth living’) is the most commonly used indicator of subjective well-being. A Japanese study has reported that subjects without a sense of *ikigai* were significantly associated with an increased risk of all-cause mortality (Sone *et al.* 2008).

In addition, we repeated the analyses after excluding all deaths that occurred within the first 3 years of follow-up, because subjects who died during this period might have been in poor health at the baseline.

Furthermore, we calculated the HRs and 95% CIs for suicide mortality by dividing the follow-up period into two separate terms (from 1990 to 1997, and from 1998 to 2008). In addition, we examined in detail potential confounding and effect modification by age and other covariates on the associations between personality scales and suicide mortality. No statistically significant interaction was observed between personality and other confounding factors for suicide mortality on a multiplicative scale (data not shown).

All statistical analyses were performed using the SAS software package, v. 9.2 (SAS Institute Inc., USA). All reported *p* values for analysis of linear trends were two-sided, and were considered statistically significant if <0.05.

## Results

**Fig. 1.**
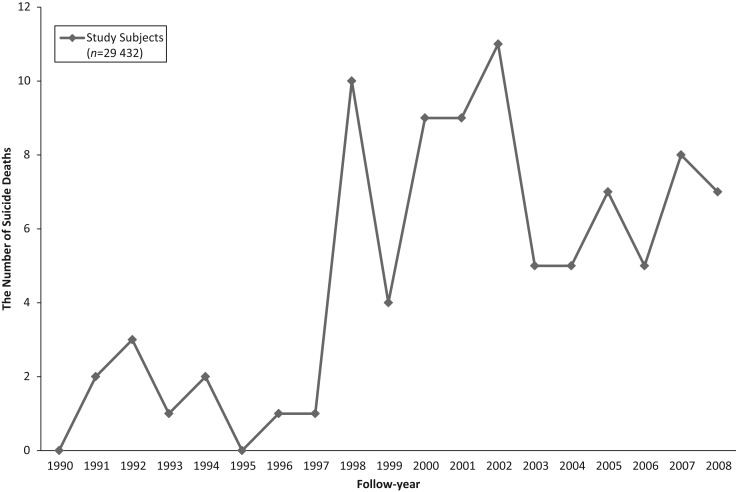
Annual trend for the number of suicide deaths 1990–2008 (The Miyagi Cohort Study).

Fig. 1 shows the annual trend for the number of suicide deaths among the study subjects (*n* = 29 432). The suicide mortality rate increased after 1998 by 6-fold relative to before 1998: from 4.6 to 27.8 per 100 000 person-years.

Baseline characteristics according to the highest and lowest categories (i.e. approximate quartiles) of each personality subscale are shown in Table 1. Higher scores for Extraversion were associated with older age, being male, being having smoked or having drunk alcohol, being overweight, being married, having *ikigai* and not having high stress. Higher scores for Neuroticism were associated with younger age, being female, having a history of hypertension or diabetes mellitus, drinking green tea less often, short sleep duration, not having *ikigai*, and having high stress. Higher scores for Psychoticism were associated with having smoked or having drunk alcohol, being regularly employed by a company more often, and being a housewife, unemployed or in other industry less often. Higher scores for Lie were associated with older age, being female, having a history of hypertension or diabetes mellitus, never having smoked or never having drunk alcohol, feeling *ikigai* more often, marrying less often, walking more often, and having high stress less often.

**Table 1.** Characteristics of the subjects according to the highest and lowest categories of four personality subscales, the Miyagi Cohort, Japan, 1990–2008

Characteristics	Personality subscales							
	Extraversion		Neuroticism		Psychoticism		Lie	
	⩽3	⩾9	⩽3	⩾8	⩽2	⩾5	⩽5	⩾10
Number of subjects	8694	6151	8495	8381	9551	6863	7177	5980
Mean age, years (s.d.)	50.8 (7.5)	51.1 (7.4)	51.4 (7.6)	50.6 (7.5)	51.9 (7.6)	49.8 (7.3)	48.0 (6.9)	54.3 (7.0)
Women (%)	53.2	47.7	47.4	54.0	65.3	32.0	39.3	57.0
Past history of diseases (%)	21.5	20.1	18.4	22.7	22.0	18.7	18.1	23.6
Smoking status (%)								
Never smoked	48.5	41.6	44.1	46.5	55.3	32.1	39.0	49.0
Smoked in the past	10.3	10.1	9.8	10.6	8.6	12.4	11.9	8.9
Currently smoking	29.5	37.4	35.2	30.3	21.7	47.5	43.0	25.6
Missing	11.7	10.9	10.9	12.6	14.4	8.0	6.1	16.5
Drinking status (%)								
Never drunk alcohol	43.3	30.0	36.0	37.4	45.8	27.2	29.2	43.2
Drank in the past	5.1	4.4	4.3	5.5	4.2	5.8	5.1	4.8
Currently drinking	42.8	57.5	51.2	48.1	39.5	60.7	61.0	39.7
Missing	8.8	8.1	8.5	9.0	10.5	6.3	4.7	12.3
BMI (kg/m^2^) (%)								
⩽18.4	3.2	1.5	1.5	3.1	2.6	2.1	2.4	2.3
18.5–24.9	69.6	62.3	63.8	68.3	67.4	65.7	67.6	66.4
⩽25.0	24.2	32.9	31.6	25.1	27.1	28.4	27.5	27.1
Missing	3.1	3.3	3.1	3.5	2.9	3.8	2.5	4.2
Green tea consumption (%)								
<1 cup/day	28.1	24.2	23.9	27.7	25.3	27.5	27.8	23.6
1–2 cups/day	21.6	20.3	21.9	21.6	20.9	21.3	23.2	20.3
⩽3 cups/day	40.0	45.4	44.0	39.6	44.0	39.7	40.6	43.5
Missing	10.3	10.1	10.2	11.1	9.8	11.5	8.4	12.6
Education level (%)								
⩽15 years	36.1	31.8	33.4	34.5	33.3	35.2	29.1	38.5
16–18 years	44.6	47.2	46.2	45.7	47.6	44.6	49.5	42.8
⩾19 years	13.9	16.3	15.2	14.1	14.3	14.1	16.9	12.7
Missing	5.4	4.7	5.2	5.7	4.8	6.1	4.5	6.0
Marital status (%)								
Living with spouse	81.6	85.4	83.3	82.9	82.9	83.2	87.0	79.2
Not living with spouse	9.8	7.6	8.2	9.3	9.2	8.6	7.7	10.0
Missing	8.6	7.0	8.5	7.8	7.9	8.2	5.3	10.8
Sleep duration (%)								
⩽6 h	20.3	20.8	16.8	24.2	21.5	20.0	22.0	17.4
7–8 h	71.0	71.0	74.0	67.7	71.0	70.1	70.8	71.6
⩾9 h	6.4	6.3	7.0	5.8	5.6	7.3	5.7	8.3
Missing	2.3	1.9	2.2	2.3	1.9	2.6	1.5	2.7
Walking (%)								
<0.5 h/day	32.1	26.7	26.3	32.5	27.9	31.7	32.9	24.8
0.5–1 h/day	22.8	24.3	24.3	22.9	24.8	22.1	24.9	22.1
>1 h/day	38.3	43.9	43.3	38.6	41.2	39.5	38.9	45.1
Missing	6.8	5.1	6.1	6.0	6.1	6.7	3.3	8.0
*Ikigai* (%)								
Having	43.6	75.9	67.8	48.2	59.4	56.2	54.6	66.6
Uncertain	48.8	20.4	27.8	44.1	35.6	36.5	40.1	27.9
Not having	4.6	1.4	1.4	4.9	2.1	4.2	3.3	1.9
Missing	3.0	2.3	3.0	2.8	2.9	3.1	2.0	3.6
Perceived mental stress (%)								
High	27.0	17.8	7.0	40.5	23.4	20.2	26.6	15.1
Medium	62.3	61.0	66.5	53.1	63.8	61.4	61.3	65.1
Low	9.1	19.7	24.9	4.6	11.3	16.3	10.8	17.6
Missing	1.6	1.5	1.6	1.8	1.5	2.1	1.3	2.2
Type of job (%)								
Employed by company	23.6	24.4	22.8	24.3	21.4	26.7	30.1	17.5
Employed by government^a^	9.9	8.8	9.3	9.5	9.5	9.2	11.7	6.8
Primary industry^b^	12.6	17.5	15.5	13.5	12.0	18.1	17.2	12.9
Self-employed, or freelance professional	19.5	19.0	21.6	18.7	19.1	20.8	15.6	25.1
Housewife, unemployed, or other	24.8	22.0	22.2	24.5	28.3	16.7	18.7	27.2
Missing	9.6	8.3	8.6	9.5	9.7	8.5	6.7	10.5

s.d., Standard deviation; BMI, body mass index.

a Medical care personnel, teacher, and civil service.

b Agriculture, fishing, and forestry.

Table 2 shows the association between personality subscales and suicide mortality during the whole study period of 1990–2008. Among the four personality traits, only Neuroticism and Psychoticism were significantly associated with suicide risk. For Neuroticism, higher category was associated with a significantly increased risk of suicide, compared with the lowest category (HR 2.04, 95% CI 1.12–3.72, *p* = 0.003 for trend). For Psychoticism, the highest category was associated with a significantly increased risk of suicide, compared with the lowest category (HR 2.30, 95% CI 1.28–4.15, *p* = 0.02 for trend). We further divided the subjects into four groups on the basis of the same range of Personality Subscale scores (0–3, 4–6, 7–9, 10–12), and obtained quite similar results (data not shown). We also modelled each Personality Subscale score as a continuous variable, and calculated the relative risk of suicide mortality for each 1-point increment of the subscale. This showed that the risk of suicide mortality was significantly associated with Neuroticism (HR 1.12, 95% CI 1.04–1.21) and Psychoticism (HR 1.15, 95% CI 1.02–1.29) but not with Extraversion or Lie. These findings remained unchanged even after excluding all deaths that occurred within the first 3 years of follow-up. Similar results were obtained among men and women, respectively.

**Table 2.** Hazard ratios and 95% confidence intervals for risk of death from suicide, the Miyagi Cohort, Japan, 1990–2008

	Personality subscale score levels				Per 1 point of score^a^	*p* trend^b^
Extraversion	⩽3	4–5	6–8	⩾9		
No. of subjects	8694	6446	8141	6151		
No. of deaths	23	25	22	20		
Person-years of follow-up	149 229	110 868	139 071	105 427		
Age and sex adjusted HR (95% CI)	1.00 (ref.)	1.47 (0.84–2.60)	1.01 (0.57–1.82)	1.16 (0.64–2.11)	1.01 (0.94–1.08)	0.92
Multivariable HR1^c^ (95% CI)	1.00 (ref.)	1.59 (0.90–2.81)	1.14 (0.63–2.06)	1.37 (0.74–2.56)	1.03 (0.96–1.10)	0.51
Multivariable HR2^d^ (95% CI)	1.00 (ref.)	1.40 (0.77–2.56)	1.19 (0.65–2.17)	1.36 (0.72–2.58)	1.03 (0.96–1.11)	0.44
Neuroticism	⩽3	4–5	6–7	⩾8		
No. of subjects	8495	6365	6191	8381		
No. of deaths	18	18	15	39		
Person-years of follow-up	146 275	109 540	105 698	143 081		
Age and sex adjusted HR (95% CI)	1.00 (ref.)	1.41 (0.73–2.71)	1.23 (0.62–2.44)	2.39 (1.37–4.18)	1.14 (1.06–1.22)	0.0003
Multivariable HR1^c^ (95% CI)	1.00 (ref.)	1.31 (0.68–2.54)	1.10 (0.55–2.20)	2.04 (1.12–3.72)	1.12 (1.04–1.21)	0.003
Multivariable HR2^d^ (95% CI)	1.00 (ref.)	1.58 (0.79–3.15)	1.21 (0.58–2.55)	2.26 (1.19–4.30)	1.12 (1.04–1.21)	0.02
Psychoticism	⩽2	3	4	⩾5		
No. of subjects	9551	7169	5849	6863		
No. of deaths	17	14	18	41		
Person-years of follow-up	164 771	123 038	100 390	116 396		
Age and sex adjusted HR (95% CI)	1.00 (ref.)	0.96 (0.47–1.95)	1.38 (0.70–2.68)	2.30 (1.28–4.13)	1.15 (1.02–1.30)	0.001
Multivariable HR1^c^ (95% CI)	1.00 (ref.)	0.97 (0.47–1.97)	1.36 (0.70–2.67)	2.30 (1.28–4.15)	1.15 (1.02–1.29)	0.002
Multivariable HR2^d^ (95% CI)	1.00 (ref.)	0.81 (0.39–1.71)	1.18 (0.59–2.36)	2.06 (1.14–3.73)	1.12 (0.99–1.27)	005
Lie	⩽5	6–7	8–9	⩾10		
No. of subjects	7177	7378	8897	5980		
No. of deaths	33	22	21	14		
Person-years of follow-up	122 423	127 046	152 811	102 315		
Age and sex adjusted HR (95% CI)	1.00 (ref.)	0.76 (0.44–1.32)	0.66 (0.38–1.16)	0.68 (0.35–1.30)	0.93 (0.86–1.01)	0.15
Multivariable HR1^c^ (95% CI)	1.00 (ref.)	0.78 (0.45–1.35)	0.70 (0.39–1.23)	0.76 (0.39–1.48)	0.94 (0.87–1.02)	0.28
Multivariable HR2^d^ (95% CI)	1.00 (ref.)	0.75 (0.43–1.32)	0.71 (0.40–1.27)	0.72 (0.36–1.45)	0.94 (0.86–1.02)	0.26

HR, Hazard ratio; CI, confidence interval.

a HRs and 95% CIs per increment of 1 point at each personality subscales were calculated by treating the personality subscale (a score of 0–12) as continuous variables.

b Linear trend tests were calculated by treating the personality subscales as continuous variables.

c HR1 was adjusted for age, sex, past histories of hypertension or diabetes mellitus (presence or absence), smoking status (never, former, currently smoking 1–19 cigarettes/day, or ⩾20 cigarettes/day), alcohol consumption (never, former, current ethanol intake of <23.0, or ≥23.0 g/day), body mass index in kg/m^2^ (⩽18.4, 18.5–24.9, or ⩾25.0), green tea consumption (<1 cup/day, 1–2 cups/day, or ⩾3 cups/day), education (in school until age ⩽15 years, age 16–18 years, or age ⩾19 years) marital status (whether or not living with spouse), sleep duration (⩽6, 7–8, or ⩾9 h/day), time spent walking (<0.5, 0.5–1, or >1 h/day), sense of life worth living (*ikigai*) (having, uncertain, or not having) and stress (high, moderate, or low).

d HR2 denotes the HR1 with deaths from all-causes in the first 3 years of follow-up excluded from the analysis.

**Fig. 2.**
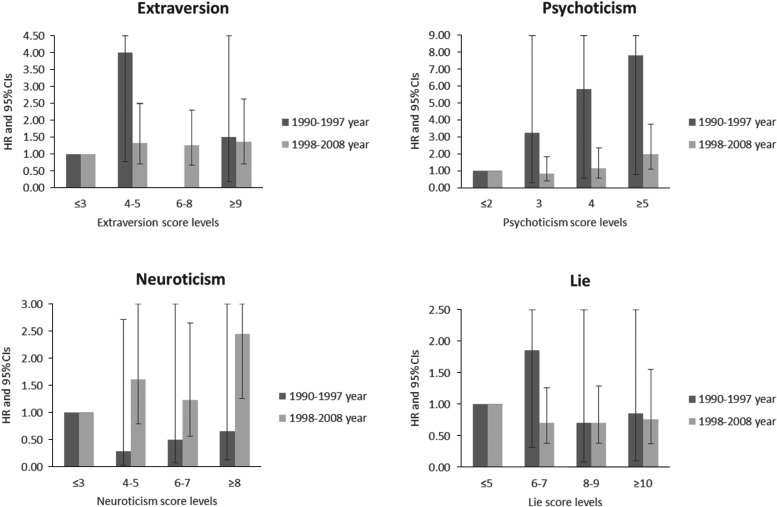
Hazard ratios for risk of death from suicide divided follow-up years into two separate terms (1990–1997, 1998–2008), the Miyagi Cohort, Japan, 1990–2008.

Table 3 shows the HRs and 95% CIs for suicide mortality for the two separate periods (from 1990 to 1997, and from 1998 to 2008). Fig. 2 graphically demonstrated the same data as for Table 3. The association between Neuroticism and suicide risk became evident only after 1998. Between 1990 and 1997, a higher Neuroticism score was associated with a non-significantly decreased risk.

**Table 3.** Hazard ratios for risk of death from suicide divided follow-up years into two separate terms (1990–1997, 1998–2008), the Miyagi Cohort, Japan, 1990–2008

1990–1997							1998–2008						
	Personality subscale score levels				Per 1 point of score^a^	*p* trend^b^		Personality subscale score levels				Per one point of score^a^	*p* trend^b^
Extraversion	⩽3	4–5	6–8	⩾9			Extraversion	⩽3	4–5	6–8	⩾9		
No. of subjects	8694	6446	8141	6151			No. of subjects	8181	6083	7627	5782		
No. of deaths	2	6	0	2			No. of deaths	21	19	22	18		
Person-years	64 111	47 582	59 915	45 372			Person-years	85 095	63 270	79 135	60 040		
Multivariable HR1^c^ (95% CI)	1.00 (ref.)	4.01 (0.77–20.83)	n.a.	1.51 (1.19–11.79)	1.00 (0.81–1.24)	0.63	Multivariable HR1^c^ (95% CI)	1.00 (ref.)	1.33 (0.71–2.49)	1.25 (0.68–2.30)	1.37 (0.71–2.63)	1.04 (0.96–1.13)	0.29
Neuroticism	⩽3	4–5	6–7	⩾8			Neuroticism	⩽3	4–5	6–7	⩾8		
No. of subjects	8495	6365	6191	8381			No. of subjects	8028	6014	5782	7849		
No. of deaths	4	1	2	3			No. of deaths	14	17	13	36		
Person-years	62 794	46 994	45 491	61 701			Person-years	83 459	62 530	60 191	81 359		
Multivariable HR1^c^ (95% CI)	1.00 (ref.)	0.29 (0.03–2.72)	0.50 (0.08–3.02)	0.66 (0.13–3.37)	1.01 (0.81–1.26)	0.65	Multivariable HR1^c^ (95% CI)	1.00 (ref.)	1.62 (0.79–3.30)	1.23 (0.57–2.65)	2.45 (1.26–4.74)	1.13 (1.04–1.23)	0.003
Psychoticism	⩽2	3	4	⩾5			Psychoticism	⩽2	3	4	⩾5		
No. of subjects	9551	7169	5849	6863			No. of subjects	9019	6738	5510	6406		
No. of deaths	1	2	3	4			No. of deaths	16	12	15	37		
Person-years	70 559	52 886	43 106	50 428			Person-years	94 187	70 134	57 269	65 950		
Multivariable HR1^c^ (95% CI)	1.00 (ref.)	3.25 (0.29–36.58)	5.85 (0.59–58.35)	7.85 (0.81–76.04)	1.49 (1.05–2.11)	0.05	Multivariable HR1^c^ (95% CI)	1.00 (ref.)	0.86 (0.41–1.83)	1.17 (0.57–2.38)	2.05 (1.11–3.78)	1.13 (0.99–1.30)	0.01
Lie	⩽5	6–7	8–9	⩾10			Lie	⩽5	6–7	8–9	⩾10		
No. of subjects	7177	7378	8897	5980			No. of subjects	6700	6958	8373	5642		
No. of deaths	2	4	2	2			No. of deaths	31	18	19	12		
Person-years	52 711	54 480	65 599	44 189			Person-years	69 694	72 547	87 188	58 111		
Multivariable HR1^c^ (95% CI)	1.00 (ref.)	1.86 (0.32–10.85)	0.71 (0.09–5.42)	0.86 (0.11–6.66)	0.92 (0.71–1.18)	0.58	Multivariable HR1^c^ (95% CI)	1.00 (ref.)	0.71 (0.39–1.27)	0.71 (0.39–1.29)	0.77 (0.38–1.56)	0.96 (0.87–1.05)	0.45

HR, Hazard ratio; CI, confidence interval.

a HRs and 95% CIs per increment of 1 point at each personality subscales were calculated by treating the personality subscale (a score of 0–12) as continuous variables.

b Linear trend tests were calculated by treating the personality subscales as continuous variables.

c HR1 was adjusted for age, sex, past histories of hypertension or diabetes mellitus (presence or absence), smoking status (never, former, currently smoking 1–19 cigarettes/day, or ⩾20 cigarettes/day), alcohol consumption (never, former, current ethanol intake of <23.0, or ⩾23.0 g/day), body mass index in kg/m^2^ (⩽18.4,18.5–24.9, or ⩾25.0), green tea consumption (<1 cup/day, 1–2 cups/day, or ⩾3 cups/day) education (in school until age ⩽15 years, age 16–18 years, or age ⩾19 years) marital status (whether or not living with spouse), sleep duration (⩽6, 7–8, or ⩾9 h/day), time spent walking (>0.5, 0.5–1, or >1 h/day), sense of life worth living (*ikigai*) (having, uncertain, or not having) and stress (high, moderate, or low).

After 1998, however, the highest category of Neuroticism became a significant factor associated with increased risk (HR 2.45, 95% CI 1.26–4.74, *p* = 0.003 for trend). For Psychoticism, the risk for suicide mortality was significantly increased at the highest category in both 1990–1997 (HR 7.85) and 1998–2008 (HR 2.05). The impact of Psychoticism on suicide risk became attenuated after 1998. For Extraversion and Lie, the association with suicide mortality after 1998 remained unchanged in comparison with that before 1998.

The above findings might be explained by the association between personality and occupation, because a previous study reported that cause-specific mortality in Japan was different among the types of job (Wada *et al.* 2012). In addition, Dupre *et al.* (2012) also reported that unemployment status is a risk of acute myocardial infarction. Thus, we considered the association between personality and types of job.

Table 1 shows the distribution of job types, and as can be seen, the distribution differed slightly according to personality. Subjects with higher Psychoticism scores were more likely to be employed by a company, and thus their employment status was less stable than in government or primary industry. However, there was no significant association between suicide mortality risk and job type. The HRs and 95% CIs were 1.05 (0.55–2.01), 1.12 (0.56–2.25), 1.26 (0.62–2.54), and 1.05 (0.48–2.31) for subjects who were employed by a company, primary industry, self-employed or freelance professional, and other (housewife, unemployed or other), respectively, when we treated subjects who were employed by the government as a reference group.

## Discussion

We investigated the association between personality and suicide risk for 19 years from 1990 to 2008 in a population-based prospective cohort study in Japan, which suffered an economic crisis in 1997–1998. We found that the suicide rate jumped in 1998, and remained high. This trend was the same as that in whole Japan (Statistics and Information Department Minister's Secretariant Ministry of Health Labour and Welfare Japan, 2011). Neuroticism and Psychoticism were significantly associated with an increased risk of suicide throughout the entire follow-up period. However, the impact of the economic crisis upon suicide risk differed between Neuroticism and Psychoticism. The suicide risk associated with Neuroticism increased after 1998, while that associated with Psychoticism decreased. The present results suggest that individuals with higher Neuroticism would be vulnerable to social stress such as economic crisis, whereas individuals with higher Psychoticism would be insensitive.

Many cross-sectional and case-control studies have reported factors associated with suicidal attempts and ideation (Park *et al.* 2010; Naragon-Gainey & Watson, 2011). The present study demonstrated for the first time the association between personality and completed suicide using a prospective approach. To our knowledge, no previous study has examined the impact of economic crisis upon the association between personality and suicide risk.

The present findings would not be explained by occupation, because occupation was not related to suicide risk in this data set. Moreover, the results cannot be explained by marital status because our data showed no association between marital status and personality.

The present finding appears to be explained by susceptibility of the subjects with a higher neuroticism score to development of depression after a major life event, which is an established risk factor for completed suicide. Many studies have shown that higher Neuroticism is associated with a higher prevalence of self-perception of mental stress. Kendler and colleagues reported that individuals with higher levels of Neuroticism were more sensitive to the depressogenic effect of adverse events than those with lower levels of Neuroticism (Kendler *et al.* 2004). In addition, Ormel *et al.* reported that stressful life events predisposed to major depression only in individuals with higher levels of Neuroticism (Ormel *et al.* 2001). Cox *et al.* suggested that Neuroticism may represent robust psychological dimensions associated with the presence post-traumatic stress disorder (PTSD; Cox *et al.* 2004). Numerous psychological autopsy studies have shown that depression is one of the strongest factors associated with completed suicides in North American and European countries, as well as in Japan (Yoshimasu *et al.* 2008; Hirokawa *et al.* 2012). Along with the above evidence, the present results can be interpreted as indicating that neuroticism was a predisposing factor for depression after the economic crisis of 1997–1998, thus causing the subsequent increase in the suicide completion.

Our study had some methodological strengths. This is the first study to investigate the interactive effects of economic crisis and personality upon suicide risk, comparing the suicide risk by personality before and after the economic crisis in 1997–1998. In addition, our sample size was sufficiently large to investigate the joint effect of economic crisis and personality upon suicide risk. This allowed us to conduct separate analyses for the periods before and after 1998.

Our study also had several limitations. First, we had no information about potential confounders, such as the prevalence of psychiatric disorders including depression, availability of social support, and blood pressure. Second, we had no information about changes in the lifestyle habits of our study subjects during the follow-up period, some misclassification of lifestyle habits could have arisen. Third, we had no individual-level information on how the economic crisis in 1997–1998 influenced members of the cohort or how much stress each member experienced. Therefore, the possibility of ecological fallacy cannot be ruled out. Fourth, we excluded 11 991 subjects out of the original cohort of 41 423 due to missing in part of answers (*N* = 8600), response by other family members (*N* = 2493), and other reasons. In order to evaluate the degree of bias resulting from exclusion of the subjects for whom answers to the EPQ-R were partly missing, we conducted ‘sensitivity analyses’ in which we imputed the missing score for each EPQ question as either ‘0’ or ‘1’ to the 8600 subjects. For both Table 2 and Table 3, all three analyses (original result, missing score imputed by 0, that imputed by 1) yielded similar results (data not shown). Therefore, we consider that the bias due to exclusion of subjects lacking answers was not sufficiently large to affect the present findings.

In conclusion, this study has demonstrated that, after the economic crisis in 1997–1998, the suicide mortality was increased only among those with higher Neuroticism but not other personality traits. Suicide mortality usually increases after major events such as economic crises and natural disasters. In this study, we have demonstrated that subjects with higher Neuroticism were at high risk of increased suicide mortality after the economic crisis in 1997–1998. This finding highlights the importance of establishing a programme for identifying vulnerable subpopulations after major events (i.e. those with higher Neuroticism) and for providing them with measures for suicide prevention such as family support, peer support, psychological consultation, early detection of depressive symptoms, and proper treatment.

## Figures and Tables

**Fig. A1.**
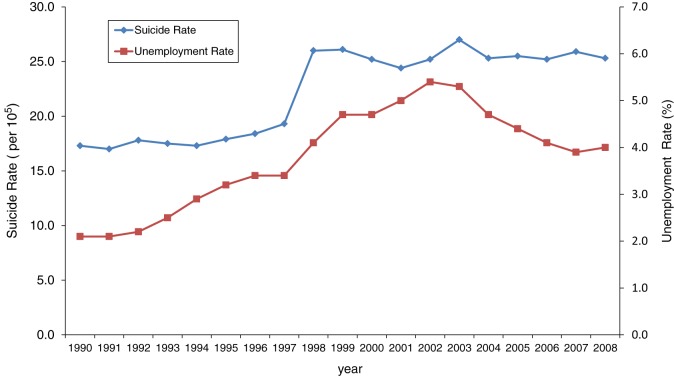
Suicide rate and unemployment rate in Japan, 1990-2008.

**Fig. A2.**
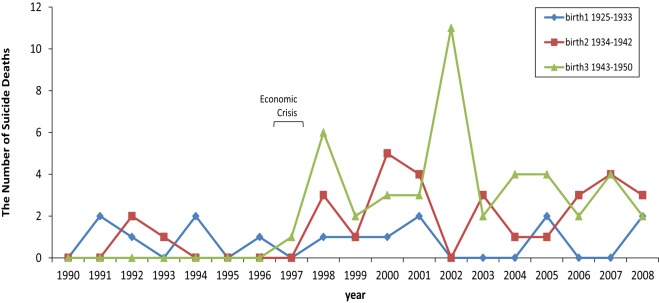
Number of suicide deaths in the subjects of Miyagi Cohort Study 1990-2008.

## Appendix

**Table A1.** Hazard ratios and 95% confidence intervals for risk of death from suicide, the Miyagi Cohort, Japan, 1990–2008

	Personality subscale score levels				*p* trend^a^
	Q1	Q2	Q3	Q4	
	0–3	4–6	7–9	10–12	
Extraversion					
No. of subjects	8694	9461	7263	4014	
No. of deaths	23	34	15	18	
Person-years	149 229	162 174	124 334	68 858	
Age- and sex-adjusted HR (95% CI)	1.00 (ref.)	1.36 (0.80–2.31)	0.78 (0.40–1.47)	1.58 (0.85–2.93)	0.82
Multivariable HR1^b^ (95% CI)	1.00 (ref.)	1.48 (0.87–2.53)	0.88 (0.45–1.71)	1.93 (1.02–3.68)	0.42
Neuroticism					
No. of subjects	8495	9579	7824	3534	
No. of deaths	18	25	25	22	
Person-years	146 275	164 128	134 151	60 041	
Age- and sex-adjusted HR (95% CI)	1.00 (ref.)	1.31 (0.72–2.40)	1.63 (0.89–2.98)	3.22 (1.73–6.01)	0.0002
Multivariable HR1^b^ (95% CI)	1.00 (ref.)	1.22 (0.66–2.26)	1.45 (0.77–2.71)	2.77 (1.40–5.49)	0.003
Psychoticism					
No. of subjects	16 720	11 469	1214	29	
No. of deaths	31	53	6	0	
Person-years	287 809	196 032	20 290	464	
Age- and sex-adjusted HR (95% CI)	1.00 (ref.)	1.95 (1.24–3.06)	1.75 (0.72–4.26)	n.s.	0.02
Multivariable HR1^b^ (95% CI)	1.00 (ref.)	1.95 (1.24–3.06)	1.64 (0.67–4.03)	n.s.	0.02
Lie					
No. of subjects	2798	7666	12 988	5980	
No. of deaths	18	29	29	14	
Person-years	47 555	131 180	223 544	102 315	
Age- and sex-adjusted HR (95% CI)	1.00 (ref.)	0.68 (0.38–1.23)	0.46 (0.25–0.84)	0.50 (0.24–1.04)	0.07
Multivariable HR1^b^ (95% CI)	1.00 (ref.)	0.71 (0.39–1.29)	0.49 (0.27–0.90)	0.58 (0.28–1.22)	0.16

HR, Hazard ratio; CI, confidence interval; n.s., non-significant.

a Linear trend tests were calculated by treating the personality subscales as continuous variables.

b HR1 was adjusted for age, sex, past histories of hypertension or diabetes mellitus (presence or absence), smoking status (never, former, currently smoking 1–19 cigarettes/day, or ⩾20 cigarettes/day), alcohol consumption (never, former, current ethanol intake of <23.0, or ⩾23.0 g/day), body mass index in kg/m^2^ (⩽18.4, 18.5–24.9, or ⩾25.0), green tea consumption (<1 cup/day, 1–2 cups/day, or ⩾3 cups/day), education (in school until age ⩽15 years, age 16–18 years, or age ⩾19 years) marital status (whether or not living with spouse), sleep duration (⩽6, 7–8, or ⩾9 h/day), time spent walking (<0.5, 0.5–1, or >1 h/day), sense of life worth living (*ikigai*) (having, uncertain, or not having) and stress (high, moderate, or low).

**Table A2.** Hazard ratios and 95% confidence intervals for risk of death from suicide, where imputed missing data as ‘0’

	Personality subscale score levels				Per 1 point of score^a^	*p* trend^b^
Extraversion	⩽3	4–5	6–8	⩾9		
No. of subjects	11 183	8295	10 465	7378		
No. of deaths	29	32	32	23		
Person-years	191 749	142 547	178 793	126 401		
Multivariable HR1^c^ (95% CI)	1.00 (ref.)	1.57 (0.94–2.60)	1.28 (0.76–2.13)	1.31 (0.74–2.32)	1.03 (0.97–1.10)	0.34
Neuroticism	⩽3	4–5	6–7	⩾8		
No. of subjects	10 943	8245	7896	10 237		
No. of deaths	21	24	24	47		
Person-years	188 347	141 775	134 847	174 521		
Multivariable HR1^c^ (95% CI)	1.00 (ref.)	1.50 (0.83–2.71)	1.52 (0.84–2.76)	2.21 (1.28–3.82)	1.12 (1.05–1.20)	<0.001
Psychoticism	⩽2	3	4	⩾5		
No. of subjects	11 782	9204	7499	8836		
No. of deaths	22	22	24	48		
Person-years	203 013	158 211	128 267	149 998		
Multivariable HR1^c^ (95% CI)	1.00 (ref.)	1.16 (0.64–2.09)	1.42 (0.79–2.55)	2.09 (1.24–3.53)	1.12 (1.01–1.25)	0.04
Lie	⩽5	6–7	8–9	⩾10		
No. of subjects	8422	9247	11 807	7845		
No. of deaths	41	27	28	20		
Person-years	143 594	159 056	202 649	134 190		
Multivariable HR1^c^ (95% CI)	1.00 (ref.)	0.73 (0.45–1.20)	0.66 (0.40–1.09)	0.79 (0.45–1.40)	0.94 (0.88–1.02)	0.12

HR, Hazard ratio; CI, confidence interval.

a HRs and 95% CIs per increment of 1 point at each personality subscale were calculated by treating the personality subscale (a score of 0–12) as continuous variables.

b Linear trend tests were calculated by treating the personality subscales as continuous variables.

c HR1 was adjusted for age, sex, past histories of hypertension or diabetes mellitus (presence or absence), smoking status (never, former, currently smoking 1–19 cigarettes/day, or ≥20 cigarettes/day), alcohol consumption (never, former, current ethanol intake of <23.0, or ⩾23.0 g/day), body mass index in kg/m^2^ (⩽18.4, 18.5–24.9, or ⩾25.0), green tea consumption (<1 cup/day, 1–2 cups/day, or ⩾3 cups/day), education (in school until age ⩽15 years, age 16–18 years, or age ⩾19 years) marital status (whether or not living with spouse), sleep duration (⩽6, 7–8, or ⩾9 h/day), time spent walking (<0.5, 0.5–1, or >1 h/day), sense of life worth living (*ikigai*) (having, uncertain, or not having) and stress (high, moderate, or low).

**Table A3:** Hazard ratios and 95% confidence intervals for risk of death from suicide, where imputed missing data as ‘1’

	Personality subscale score levels				Per 1 point of score^a^	*p* trend^b^
Extraversion	⩽3	4–5	6–8	⩾9		
No. of subjects	10 645	8201	10 675	7800		
No. of deaths	29	30	33	24		
Person-years	182 465	141 034	182 536	133 455		
Multivariable HR1^c^ (95% CI)	1.00 (ref.)	1.43 (0.86–2.40)	1.25 (0.75–2.08)	1.24 (0.71–2.18)	1.03 (0.96–1.09)	0.43
Neuroticism	⩽3	4–5	6–7	⩾8		
No. of subjects	10 307	8043	7943	11 028		
No. of deaths	20	25	21	50		
Person-years	177 583	138 128	135 877	187 902		
Multivariable HR1^c^ (95% CI)	1.00 (ref.)	1.59 (0.88–2.87)	1.32 (0.71–2.45)	2.19 (1.26–3.80)	1.11 (1.04–1.19)	0.001
Psychoticism	⩽2	3	4	⩾5		
No. of subjects	11 960	9176	7426	8759		
No. of deaths	22	23	24	47		
Person-years	206 121	157 550	127 369	148 449		
Multivariable HR1^c^ (95% CI)	1.00 (ref.)	1.23 (0.69–2.22)	1.45 (0.81–2.61)	2.09 (1.24–3.54)	1.12 (1.00–1.24)	0.04
Lie	⩽5	6–7	8–9	⩾10		
No. of subjects	8729	9341	11 529	7722		
No. of deaths	41	28	29	18		
Person-years	148 675	160 531	198 110	132 174		
Multivariable HR1^c^ (95% CI)	1.00 (ref.)	0.78 (0.48–1.27)	0.72 (0.44–1.19)	0.74 (0.41–1.33)	0.95 (0.88–1.02)	0.13

HR, Hazard ratio; CI, confidence interval.

a HRs and 95% CIs per increment of 1 point at each personality subscales were calculated by treating the personality subscale (a score of 0–12) as continuous variables.

b Linear trend tests were calculated by treating the personality subscales as continuous variables.

c HR1 was adjusted for age, sex, past histories of hypertension or diabetes mellitus (presence or absence), smoking status (never, former, currently smoking 1–19 cigarettes/day, or ⩾20 cigarettes/day), alcohol consumption (never, former, current ethanol intake of <23.0, or ⩾23.0 g/day), body mass index in kg/m^2^ (⩽18.4, 18.5–24.9, or ⩾25.0), green tea consumption (<1 cup/day, 1–2 cups/day, or ⩾3 cups/day), education (in school until age ⩽15 years, age 16–18 years, or age ⩾19 years) marital status (whether or not living with spouse), sleep duration (⩽6, 7–8, or ⩾9 h/day), time spent walking (<0.5, 0.5–1, or >1 h/day), sense of life worth living (*ikigai*) (having, uncertain, or not having) and stress (high, moderate, or low).
